# Variability of spatial temporal gait parameters and center of pressure displacements during gait in elderly fallers and nonfallers: A 6-month prospective study

**DOI:** 10.1371/journal.pone.0171997

**Published:** 2017-02-27

**Authors:** Zdenek Svoboda, Lucia Bizovska, Miroslav Janura, Eliska Kubonova, Katerina Janurova, Nicolas Vuillerme

**Affiliations:** 1 Department of Natural Sciences in Kinanthropology, Faculty of Physical Culture, Palacky University Olomouc, Olomouc, Czech Republic; 2 IT4 Innovations National Supercomputing Center, VSB-Technical University of Ostrava, Ostrava, Czech Republic; 3 Univ. Grenoble-Alpes, EA AGEIS, La Tronche, France; 4 Institut Universitaire de France, Paris, France; Center for BrainHealth, University of Texas at Dallas, UNITED STATES

## Abstract

Considering that most of the falls in elderly population arise during walking, tests derived from walking performance would be desirable for comprehensive fall risk assessment. The analysis of spatial temporal parameters and the center of pressure displacement, which represents the interaction between the human body and the ground, would be beneficial. The aim of this study was to compare spatial temporal gait parameters and their variability and the variability of the center of pressure displacement between elderly fallers and nonfallers during gait at self-selected, defined and fast speeds. A prospective study design was used. At the baseline, measurements of ground reaction force during gait at self-selected, defined and fast walking speeds by two force plates were performed. In addition, the Tinetti balance assessment tool, the Falls Efficacy Scale-International and the Activities-Specific Balance Confidence Scale were used. Mean and coefficient of variation of spatial temporal gait parameters and standard deviations of center of pressure displacement during loading response, midstance, terminal stance and preswing phases were calculated. Comparison of the fallers and nonfallers exhibited no significant difference in clinical tool, scales or spatial temporal parameters. Compared to nonfallers’ increased variability of walking speed at self-selected and defined speed, step width at fast walking speed and center of pressure displacement during preswing phase in medial-lateral directions at defined walking speed was found in fallers. However, application of the Holm-Bonferroni procedure for multiple comparisons exhibited no significant effect of group in any of the gait parameters. In general, our study did not observe an effect of group (fallers vs. nonfallers) on variability of spatial temporal parameters and center of pressure movement during gait. However, walking speed, step width as well as standard deviation of COP displacement in the medial-lateral direction during preswing exhibited a certain potential for distinguishing between elderly fallers and nonfallers.

## Introduction

In the scientific community, there is a growing body of research investigating procedures to determine fall risk. Clinical balance tests, such as the Berg Balance Scale [1], the Functional Reach Test [2], and the Timed ‘Up & Go’ test [2], are insufficient to predict fallers in the elderly population.

Numerous studies have used an assessment of postural stability during standing with the assumption that postural stability has a direct relationship to fall history even though most falls are associated with movement activities. Because of this, results of studies focused on the relationship between postural sways and fall risk are conflicting. Some authors confirmed that postural stability measures can determine fallers and nonfallers in healthy older adults. Merlo et al. [3] showed that the mean position of the center of pressure (COP) movement in the anterior-posterior direction and the confidence ellipse area during standing were associated with the fall-history in both open and closed eyes conditions. In contrast, some authors reported no significant difference in postural stability measures between groups of fallers and nonfallers [2].

Significant differences between elderly fallers and nonfallers during standing were observed especially in more challenging conditions such as stance in narrow base of support [4]. In addition, the ability of postural stability measures to predict falls is sufficient only for indoor fallers [5]. Similarly, traditional sway parameters were significantly different only for fallers with serious injury. Fallers without serious injuries did not significantly differ compared to nonfallers for these variables [6]. A study by Brauer et al. [1] suggests that measures of COP motion in quiet stance had a poor ability to predict fallers, although they could correctly identify most nonfallers.

Considering that walking is the most frequently cited cause of falls [7] it is clear that balance assessment during walking would be beneficial for the completion of a possible fall risk assessment. Scientific studies showed that temporal-spatial variables and their variability were able to predict fall risk [8–11]. Variability of stride time as well as variability of stance time, swing time and relative stance time were significantly higher in fallers in comparison with nonfallers [8–10]. Other factors that can predict fall risk would require nonlinear methods. Hamacher et al. [8] showed the suitability of using orbital stability measures (Floquet multipliers) applied to gait. The potential of gait variability for fall risk prediction was also confirmed by König et al. [11]. They tested three functional domains (using a total of 92 measures) including muscular control, standing balance, and mean and variability of gait performance. The results suggest that temporal variability and mean spatial parameters of gait are the only functional components among all the tested measures that differentiate fallers from nonfallers, and could therefore show efficacy in clinical screening programs for assessing the risk of first-time falling [11].

Although several studies showed the potential of spatial temporal parameters to discriminate between fallers and nonfallers, the recent review by Mortaza et al. [12] concludes that spatial temporal parameters derived from level walking are not sufficient reliable predictors for falls. One of the possible reasons of insufficient predictability is because of various gait speeds between subjects or the fact that all of these variables consider whole stride or stance. In the various phases of the gait cycle, the human body executes different functional tasks, thus assessment of variability in various gait phases would provide new insight into assessment of fall related variables during gait. For this, an assessment using force plates seems to be suitable, because they are commonly used for gait analysis in many observed groups across age, health state or disease. The purpose of the study was based on the assumption that COP movement represents interactions between the human body and the ground, which is a potential fall interface. It was shown that variability of COP movement was significantly higher in the older group for the medial-lateral direction during loading response, midstance and preswing and for the anterior-posterior direction for preswing [13, 14]. However, we did not find any study that observed the relationship between COP movement variability during gait and fall risk in older adults.

The aim of this study was to compare spatial-temporal gait parameters and variability of center of pressure displacement between elderly fallers and nonfallers during gait at self-selected, defined and fast speeds.

## Materials and methods

### Participants

Participants were recruited from local senior clubs and the University of the Third Age. Inclusion criteria for participation in the study were age (60 years old and older), the ability to walk without an assistive device, and the ability to stand unassisted without any support during common everyday activities. Exclusion criteria were neurological or vestibular disease and surgery in lower limbs or spine during the last two years. The research sample consisted of 125 subjects, 101 women and 24 men. Basic sample characteristics were (mean ± standard deviation): age 70.6 ± 6.5 years, body height 163.0 ± 7.7 cm, body weight 75.8 ± 13.6 kg and body mass index 28.4 ± 4.6 kg.m^–2^. The study was approved by the Ethics Committee of the Faculty of Physical Culture, Palacky University Olomouc under no. 24/2014. All participants provided written informed consent.

### Methods

Baseline measurements included measurement of ground reaction force, COP trajectory during gait by two force plates (9286 AA, Kistler Instrumente AG, Winterthur, Switzerland), the Tinetti balance assessment tool [15], the Falls Efficacy Scale-International (FES-I) [16], and the Activities-Specific Balance Confidence (ABC) Scale [17]. The Tinetti balance assessment tool is a clinical test for assessing both static balance and gait. Maximal scores are 12 points for gait, 16 for balance and 28 in total. A total score larger or equal to 24 is considered as low risk of falling. The FES-I questionnaire assesses fear of falling; we used a version translated into Czech by Reguli and Svobodová [18] in accordance with the methodology of the Ten Step Translation Protocol recommended of Prevention of Falls Network Earth. The total score ranges from 16 (not at all concerned about the possibility of falling during all activities) to 64 points (very concerned about the possibility of falling during all activities). The ABC scale assesses the level of confidence in performing activities of daily living without losing balance or becoming unsteady. A maximal score is 100%. A Czech version of the ABC Scale was prepared by authors of this article. The translation process followed the recommendations of the World Health Organization for translation and adaptation of instruments [19].

### Gait analysis

Participants performed barefoot walking on the 10 m walkway with 2 force plates (sample rate 200 Hz) installed in series in the middle of a walkway to record middle gait cycles. The force plate for left limb was followed by the force plate for right limb. Participants executed between 2 to 5 trials for familiarization and 5 measured trials at each self-selected, defined and fast speed. First, self-selected speed was studied because defined or fast speeds would disturb natural gait performance. Then, walking by defined speed followed. It was used to ensure comparability between subjects and trials for assessment of variables derived from the center of pressure movement. Participants were asked to match a predefined speed of gait of 4 km.h^-1^ (1.11 m.s^-1^) with ± 10% tolerance (1.00 to 1.22 m.s^-1^). It is consistent with value of 1.13 m.s^-1^, which was presented as the mean walking speed for 70 to 79 years old women in the scientific literature [20]. Walking at a fast speed was tested at the end of the experimental session because possible fatigue would influence walking at self-selected and defined speeds. For gait at a fast speed, the participants were asked to walk as fast as possible. Gait speed was determined by two photocell gates (FiTRO Light Gates, Fitronic, Bratislava, Slovakia) placed in front of and behind the force plates to capture the time during recorded gait cycles.

### Fall history assessment

Fall history observation followed recommendations of the Prevention of Falls Network Europe and Outcomes Consensus Group [21]. A fall was defined as “an unexpected event in which the participants come to rest on the ground, floor, or lower level”[21]. Falls were recorded for 6 months using prospective daily recording with a telephone interview every two weeks. Participants were asked “In the past two weeks, have you had any fall including a slip or trip in which you lost your balance and landed on the floor or ground or lower level?”. During the telephone interview, participants were also asked for the circumstances during with they fall. Falls associated with a sport activity such as cycling or skating were excluded.

### Data processing

Ground reaction force and COP position curves were filtered using a 2^nd^ order bidirectional Butterworth low-pass filter with a cut-off frequency of 30 Hz [22]. The stance phase was determined as the time interval during which the vertical ground reaction force exceeded 5% of the subject’s body weight [23].

Basic spatial temporal parameters were also derived from the center of pressure movement measured by force plates: step length = anterior-posterior (AP) distance between COP position during initial contact of left and right limbs, step width = medial-lateral (ML) distance between COP position during initial contact of left and right limbs, step time = time interval between left and right initial contacts, walking speed = step length / step time. In the case of independent values for left and right limb, the values were averaged. For all spatial temporal parameters, mean and coefficient of variation (CV = SD/Mean * 100%) from five trials were calculated and then used for analysis.

**Fig 1:**
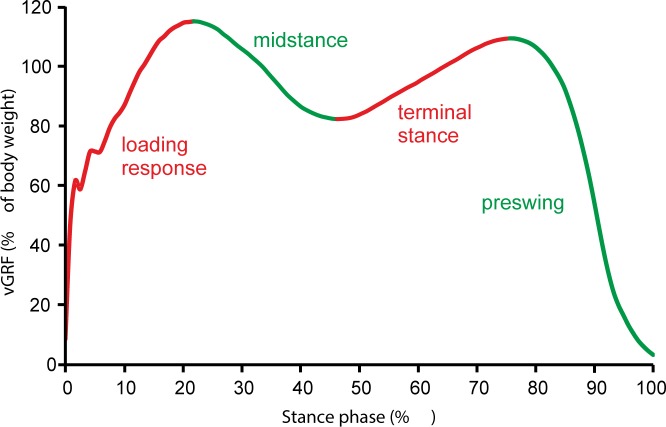
Subphases of the gait stance phase. vGRF–vertical ground reaction force.

For COP analysis, the stance phase was divided into four time intervals that corresponded to loading response, midstance, terminal stance and preswing (Fig 1). This division was decided according to the behavior of the vertical component of the ground reaction force [13, 14]: loading response = time interval between initial contact and first vertical peak, midstance = time interval between first vertical peak and minimal vertical force, terminal stance = time interval between minimal vertical force and second vertical peak, preswing = time interval between second vertical peak and toe off. In each of these intervals, COP movement variability was computed as a standard deviation of COP displacement for each direction (ML and AP) for both limbs and then averaged. Computations were performed using custom-written Matlab (MATLAB R2014b, Mathworks, Inc., Natick, MA, USA) algorithms. For statistical analysis, mean values from five trials were used.

### Statistics

The data distribution of all measured parameters was tested by the Kolmogorov-Smirnov test. In some cases a non-normal data distribution was found and therefore, nonparametric procedures were applied for all parameters. Basic characteristics, outputs from clinical balance tools, mean values and variability of spatial temporal parameters similarly as variability of COP displacement parameters in various gait cycle phases were compared between fallers and nonfallers by a Mann-Whitney U test. Effect size was determined as r = Z/√N, where Z is standardized value of the Mann-Whitney U test and N is the total number of the samples. Values 0.1 ≤ r < 0.3 were considered as small, 0.3 ≤ r < 0.5 as medium and r ≥ 0.5 as a large effect [24]. Comparison of frequency of men and women in observed groups was performed by a chi squared test.

We applied a Holm-Bonferroni correction to counteract the problem of multiple comparisons [25]. A correction factor was used for each walking speed in the three groups of parameters: spatial temporal parameters (smallest p-value = 0.05/4 = 0.0125), variability of spatial temporal parameters (smallest p-value = 0.05/4 = 0.0125), and COP movement variability (smallest p-value = 0.05/8 = 0.00625).

## Results

After fall history assessment, participants were classified as fallers (n = 31) if they fell during the observed period at least one time [1, 5, 9, 10] (the highest number of falls was 4) or nonfallers (n = 94) if they did not have any documented falls. The two groups were comparable with regards to age, body height, body weight and body mass index (Table 1). Gender ratio (relative number of women) was also similar (nonfallers: 79.8% of women, fallers 86.7% of women, p = 0.399). Finally, we did not find any significant difference between fallers and nonfallers for the Tinetti balance assessment tool, the Falls Efficacy Scale-International and the Activities-specific Balance Confidence Scale.

**Table 1:** Basic characteristics and clinical balance parameters of elderly nonfallers and fallers.

Parameter	Nonfallers (n = 94)			Fallers (n = 30)			Significance	
	Mean	Mdn	SD	Mean	Mdn	SD	p	r
Age (years)	70.4	69.7	6.6	70.9	70.5	6.2	0.661	0.040
Body height (cm)	163.4	163.0	7.6	161.5	161.9	8.1	0.230	0.108
Body weight (kg)	76.6	74.4	14.4	73.1	73.9	11.2	0.289	0.096
Body Mass Index (kg.m^-2^)	28.5	28.3	4.7	27.9	27.0	4.5	0.418	0.073
Tinetti balance	15.8	16.0	0.7	15.7	16.0	0.6	0.385	0.114
Tinetti gait	11.9	12.0	0.4	11.7	12.0	0.7	0.441	0.106
Tinetti total score	27.6	28.0	0.9	27.4	28.0	1.0	0.203	0.139
ABC	86.8	90.0	11.4	87.7	92.2	11.2	0.632	0.043
FES-I	23.1	21.5	5.7	22.6	21.0	5.1	0.717	0.033

Mdn–median, SD–standard deviation, ABC–Activities-specific Balance Confidence Scale, FES-I–Falls Efficacy Scale-International, p–significance level, r–effect size.

Spatial temporal gait parameters variables showed no significant difference between elderly fallers and nonfallers for the three walking speeds (Table 2). Assessment of variability of spatial temporal gait parameters showed significantly increased variability of walking speed at self-selected and defined speed and step width at fast speed in fallers compared to nonfallers (Table 3).

**Table 2:** Spatial temporal gait parameters at self-selected, defined and fast speeds in elderly nonfallers and fallers.

Walking speed	Parameter	Nonfallers (n = 94)			Fallers (n = 30)			Significance	
		Mean	Mdn	SD	Mean	Mdn	SD	p	r
**Self-selected**	Step length (m)	0.590	0.582	0.053	0.576	0.568	0.066	0.212	0.112
** **	Step width (m)	0.103	0.103	0.031	0.095	0.098	0.026	0.241	0.106
** **	Step time (s)	0.528	0.523	0.051	0.528	0.526	0.052	0.970	0.003
** **	Walking speed (m/s)	1.13	1.11	0.15	1.11	1.14	0.18	0.674	0.038
**Defined**	Step length (m)	0.587	0.586	0.037	0.581	0.575	0.046	0.351	0.084
** **	Step width (m)	0.100	0.101	0.032	0.097	0.091	0.027	0.363	0.082
** **	Step time (s)	0.530	0.527	0.039	0.521	0.527	0.036	0.268	0.100
** **	Walking speed (m/s)	1.11	1.10	0.06	1.12	1.13	0.07	0.398	0.076
**Fast**	Step length (m)	0.659	0.654	0.064	0.648	0.632	0.069	0.253	0.103
** **	Step width (m)	0.104	0.106	0.031	0.096	0.094	0.024	0.118	0.141
** **	Step time (s)	0.443	0.443	0.040	0.427	0.431	0.036	0.055	0.172
** **	Walking speed (m/s)	1.50	1.47	0.20	1.53	1.55	0.22	0.448	0.068

Mdn–median, SD–standard deviation, p–significance level, r–effect size.

**Table 3:** Variability of spatial temporal gait parameters (CV) at self-selected, defined and fast speeds in elderly nonfallers and fallers.

Walking speed	Parameter	Nonfallers (n = 94)			Fallers (n = 30)			Significance	
		Mean	Mdn	SD	Mean	Mdn	SD	p	r
Self-selected	Step length	3.1	2.8	1.5	3.1	3.0	1.3	0.828	0.020
	Step width	24.3	20.3	14.7	23.7	21.3	10.5	0.521	0.058
	Step time	3.5	2.9	2.2	4.1	3.8	2.2	0.056	0.172
	Walking speed	**5.0**	**4.6**	**3.0**	**5.9**	**6.1**	**2.7**	**0.020**	**0.208**
Defined	Step length	3.0	2.8	1.4	3.3	3.1	1.4	0.258	0.102
	Step width	26.3	21.6	16.2	24.6	21.8	12.3	0.951	0.006
	Step time	3.7	3.4	1.7	4.1	3.8	2.2	0.398	0.076
	Walking speed	**5.0**	**4.6**	**2.1**	**6.1**	**5.3**	**2.8**	**0.048**	**0.177**
Fast	Step length	3.3	3.0	1.9	3.7	3.1	2.8	0.657	0.040
	Step width	**22.7**	**19.8**	**15.9**	**27.7**	**23.0**	**15.2**	**0.030**	**0.194**
	Step time	3.6	3.3	1.9	3.8	3.5	2.1	0.761	0.028
	Walking speed	4.6	4.3	2.4	5.2	4.5	2.9	0.336	0.087

Mdn–median, SD–standard deviation, p–significance level, r–effect size, bold values show significant difference.

An analysis of variability of COP displacements in various stance phase subphases showed only one significant difference between fallers and nonfallers at defined walking speed. The variability of COP displacement in the medial-lateral direction during preswing was significantly higher for fallers than for nonfallers (Table 4).

**Table 4:** Variability of COP displacement (mm) in loading response, midstance, terminal stance and preswing at self-selected, defined and fast speeds in elderly nonfallers and fallers.

Walking speed	Phase	Direction	Nonfallers (n = 94)			Fallers (n = 30)			Significance	
			Mean	Mdn	SD	Mean	Mdn	SD	p	r
Self-selected	LR	ML	3.20	2.69	2.23	3.11	2.77	1.29	0.495	0.062
		AP	5.03	4.19	3.75	5.36	5.43	2.24	0.067	0.164
	MSt	ML	0.16	0.16	0.05	0.16	0.15	0.04	0.431	0.071
		AP	0.47	0.40	0.22	0.45	0.39	0.19	0.734	0.031
	TSt	ML	0.15	0.14	0.04	0.15	0.15	0.05	0.792	0.024
		AP	0.58	0.57	0.21	0.56	0.49	0.27	0.286	0.096
	PSw	ML	0.87	0.73	0.70	0.99	0.78	0.73	0.391	0.077
		AP	2.37	1.91	2.29	3.16	2.08	4.45	0.529	0.057
Defined	LR	ML	3.10	2.63	2.21	3.11	3.01	1.25	0.236	0.107
		AP	5.18	4.22	3.98	6.03	5.25	4.40	0.076	0.160
	MSt	ML	0.16	0.15	0.05	0.15	0.15	0.04	0.603	0.047
		AP	0.44	0.41	0.14	0.43	0.40	0.11	0.887	0.013
	TSt	ML	0.15	0.14	0.04	0.15	0.15	0.06	0.752	0.029
		AP	0.57	0.57	0.19	0.57	0.51	0.25	0.624	0.044
	PSw	ML	**0.85**	**0.70**	**0.68**	**1.14**	**0.88**	**1.01**	**0.045**	**0.180**
		AP	2.22	1.86	2.13	3.59	2.02	6.08	0.170	0.123
Fast	LR	ML	3.91	3.53	2.24	4.44	3.66	2.09	0.221	0.110
		AP	8.06	6.33	9.45	8.54	7.20	8.15	0.579	0.050
	MSt	ML	0.23	0.22	0.08	0.25	0.22	0.10	0.502	0.061
		AP	0.97	0.82	0.51	1.06	0.87	0.65	0.761	0.028
	TSt	ML	0.17	0.16	0.06	0.17	0.18	0.06	0.674	0.038
		AP	0.69	0.62	0.29	0.81	0.67	0.46	0.540	0.055
	PSw	ML	1.20	0.89	0.86	1.37	1.16	0.78	0.141	0.132
		AP	3.33	2.46	3.92	3.24	2.81	1.36	0.129	0.136

Mdn–median, SD–standard deviation, LR–loading response, MSt–midstance, TSt–terminal stance, PSw–preswing, ML–medial-lateral, AP–anterior-posterior, p–significance level, r–effect size, bold values show significant difference.

Application of the Holm-Bonferroni procedure for multiple comparisons showed no significant effect of group in all cases (spatial temporal parameters, variability of spatial temporal parameters, and COP movement variability).

## Discussion

The objective of this study was to compare spatial-temporal gait parameters and variability of center of pressure displacement between elderly fallers and nonfallers during gait at self-selected, defined and fast speeds. Naturally, the results of fall risk studies would be influenced by many factors including recruitment strategy or characteristics of participants (e.g., demographic, health-related, behavioral features, level of physical activity, etc.). In the present study, we asked participants from all seniors clubs in the Olomouc city (approximately 100 thousand inhabitants) and students at the University of Third Age. Thus, we expected that the distribution of participants was uniform across the city. The mean age of our participants (70.5 ± 6.5 years) is comparable [1, 5] or lower [4, 6, 10, 26] to similar studies.

We also measured balance confidence, which would be able to distinguish between older individuals at different levels of functional mobility [15]. Activities-specific to the Balance Confidence Scale are considered more appropriate for assessing seniors at various levels of functioning, particularly more active persons, while the Falls Efficacy Scale is more appropriate for assessing balance confidence in more frail seniors [17]. Although we used both of these scales, we did not find any significant difference between the observed groups. Average values of these tests suggest very good balance confidence of our participants. In the Tinetti balance assessment tool, we also did not find any significant differences. In addition, the mean values of scores showed low risk of falls [27] in both faller and nonfaller groups.

The basic approach in gait assessment represents spatial temporal parameters and their variability. It was found that slower gait speed and other basic spatial temporal variables such as swing phase time and double-support time can serve as fall risk predictors [28]. Conversely, in our study, we did not find any significant difference across all speeds.

In the scientific literature, variability of spatial temporal gait parameters has been shown to differ between elderly fallers and nonfallers. More precisely, it was reported that variability of stride, stance phase or swing phase duration and variability of stride length can distinguish between fallers and nonfallers [9, 10, 28]. At the self-selected speed, the movement of the body represents natural gait performance, and it resulted in our study in an increased walking speed in fallers compared to nonfallers. Defined speed could be useful for elimination of the speed effect. Fallers showed greater step width at the fast gait speed, which can be associated with a higher risk of cognitive decline [29].

Our results showed that differences between elderly fallers and nonfallers could be influenced by speed of the walking task. In our study, we found increased variability of walking speed for defined and self-selected walking speeds and increased step width variability for fast walking speed in fallers. However, the effect size of these differences was low, and the Bonferroni-Holm corrected multiple comparisons showed no significant effect of group.

When comparing COP displacements during gait between fallers and nonfallers, we found higher variability in the medial-lateral direction during preswing. The preswing phase is characterized by rapid transfer of body weight to the opposite limb [30]. Thus, association with following the opposite limb loading response is possible. Increased variability of movement during this phase was also presented by Barak et al. [31]. They reported that fallers are characterized by a larger standard deviation in hip extension peak compared with the nonfallers. An age effect of COP variability was observed by Bizovska et al. [13]. In addition to higher variability in medial lateral direction during preswing in older subjects, they also found increased COP variability in the medial-lateral direction during loading response and midstance and in the anterior-posterior direction during preswing in older compared to younger subjects. In our study only one significant difference (p = 0.045) was found, but after applying the correction for multiple comparisons (8 parameters), no significant effect of group was found. This could be related to the fact that both groups had high functioning levels. Due to the multifactorial nature of falls in the community dwelling elderly population, the currently used clinical and laboratory tests could not predict fall likelihood [1]. Therefore, it may be useful to find methods more capable of distinguishing between fallers and nonfallers. Our results suggest that COP variability assessment could be one of these methods. Regression analysis of these variables together with other fall risk predictors could be beneficial.

There are some limitations to be considered in this study. Most of the participants were women, because they were more willing to participate in the experiment. However, the gender ratio was comparable in the groups of fallers and nonfallers. Furthermore, our definition of fallers was limited. The elderly individual that has been fallen only once in the last six months may not be a “typical” faller. Despite this fact in the scientific literature subjects with 1 fall are commonly considered as “fallers” [1, 5, 9, 10]. In addition, falls during sporting activities which could be considered as situations with high balance demands were excluded from the analysis. Another limitation is number of trials used to assess gait performance. Due to the high number of tests and possible fatigue of participants, we observed only five trials for each walking speed per participant.

## Conclusions

Overall, our study did not reveal an effect of group (fallers vs. nonfallers) on variability in spatial temporal parameters and center of pressure movement during gait. However, walking speed, step width as well as standard deviation of COP displacement in the medial-lateral direction during preswing showed a potential for distinguishing between elderly fallers and nonfallers. The low number of significant differences between gait characteristics (spatio-temporal gait parameters, variability of these parameters, and variability of COP displacement) measured in elderly fallers and nonfallers may be partly explained by the participants’ characteristics. The elderly individuals involved in the present study were independent, community dwelling, and involved in social participation. Clinical assessments further indicated high levels of physical functioning and very good balance confidence in both elderly fallers and nonfallers groups, which could have attenuated the effects on gait. Along these lines, to further confirm the present findings and to extend the scope of our results, additional prospective studies involving older adults with lower level physical functioning and a higher risk of falling for a longer period of time are needed.

## Supporting information

S1 Dataset This is the dataset. (XLSX)
